# Neurodegeneration with brain iron accumulation: update on pathogenic mechanisms

**DOI:** 10.3389/fphar.2014.00099

**Published:** 2014-05-07

**Authors:** Sonia Levi, Dario Finazzi

**Affiliations:** ^1^Proteomic of Iron Metabolism, Vita-Salute San Raffaele UniversityMilano, Italy; ^2^San Raffaele Scientific InstituteMilano, Italy; ^3^Department of Molecular and Translational Medicine, University of BresciaBrescia, Italy; ^4^Spedali Civili di BresciaBrescia, Italy

**Keywords:** brain, iron, neurodegeneration, NBIA disorders, oxidative stress, pathogenesis

## Abstract

Perturbation of iron distribution is observed in many neurodegenerative disorders, including Alzheimer’s and Parkinson’s disease, but the comprehension of the metal role in the development and progression of such disorders is still very limited. The combination of more powerful brain imaging techniques and faster genomic DNA sequencing procedures has allowed the description of a set of genetic disorders characterized by a constant and often early accumulation of iron in specific brain regions and the identification of the associated genes; these disorders are now collectively included in the category of neurodegeneration with brain iron accumulation (NBIA). So far 10 different genetic forms have been described but this number is likely to increase in short time. Two forms are linked to mutations in genes directly involved in iron metabolism: neuroferritinopathy, associated to mutations in the *FTL* gene and aceruloplasminemia, where the *ceruloplasmin* gene product is defective. In the other forms the connection with iron metabolism is not evident at all and the genetic data let infer the involvement of other pathways: *Pank2*, *Pla2G6*, *C19orf12*, *COASY*, and *FA2H* genes seem to be related to lipid metabolism and to mitochondria functioning, *WDR45* and *ATP13A2* genes are implicated in lysosomal and autophagosome activity, while the *C2orf37* gene encodes a nucleolar protein of unknown function. There is much hope in the scientific community that the study of the NBIA forms may provide important insight as to the link between brain iron metabolism and neurodegenerative mechanisms and eventually pave the way for new therapeutic avenues also for the more common neurodegenerative disorders. In this work, we will review the most recent findings in the molecular mechanisms underlining the most common forms of NBIA and analyze their possible link with brain iron metabolism.

## INTRODUCTION

The recent advance in magnetic resonance imaging (MRI) techniques and the increased ability to identify causative genes led to the recognition of a new group of disorders named neurodegeneration with brain iron accumulation (NBIA). It is a set of degenerative extrapyramidal monogenic disorders with radiological evidence of focal accumulation of iron in the brain, usually in the basal ganglia (Yoshida et al., 1995; Gregory et al., 2009; Gregory and Hayflick, 2011). They are characterized by early or late onset, with the main symptoms associated to problems in the movement, spasticity, and cognitive impairment. The diagnosis of NBIA is made on the basis of the combination of representative clinical features along with MRI evidence of iron accumulation (Kruer and Boddaert, 2012). Imaging details can be so specific to orient the successive genetic analysis required to include the disease in a genetically confirmed category or in the idiopathic group, that still represent about 30% of all cases (Dusek et al., 2013).

Different disorders were grouped under this category (**Table 1**): neuroferritinopathy, aceruloplasminemia, pantothenate kinase-associated neurodegeneration (PKAN), phospholipase 2, group VI-associated neurodegeneration (PLAN), mitochondrial membrane protein-associated neurodegeneration (MPAN), fatty acid hydroxylase-associated neurodegeneration (FAHN), β-propeller protein-associated neurodegeneration (BPAN), and two other forms, the Kufor–Rakeb disease and the Woodhouse–Sakati syndrome, which are not always associated to brain iron accumulation. Very recently a new form was identified. Exome sequencing revealed the presence of recessive missense mutations in the COASY gene, encoding coenzyme A (CoA) synthase, in one NBIA-affected subject and confirmed in a second unrelated patient (Dusi et al., 2014). The authors proposed COASY protein-associated neurodegeneration (CoPAN) as the name to classify this disorder. The main characteristics of the different types of genetic NBIA syndromes are summarized in **Table 1**. They show variable incidence, and, from an epidemiological point of view, it is also important to note that these diseases are extremely rare and some are generally confined to specific populations (Dusek and Schneider, 2012, 2013).

**Table 1:** List of the neurodegeneration with brain iron accumulation disorders.

Human disorder	Brain regions interested by iron deposition	Symptomatology
Aceruloplasminemia	Dentate nucleus, globus pallidus, putamen, caudate	Diabetes, anemia, dementia, dystonia, dysarthria
Neuroferritinopathy; hereditary ferritinopathy; NBIA type III	Dense ferritin-Fe spheroid inclusions in dentate nuclei, globus pallidus, putamen, caudate, thalamus, and red nuclei	Dementia, dystonia, dysarthria in some cases cognitive decline
Pantothenate kinase-associated neurodegeneration (PKAN, NBIA type I, Hallervorden–Spatz syndrome)	Globus pallidus (eye of the tiger)	Dystonia, with predominant oro-lingual-mandibular involvement, and spasticity
Phospholipase 2, group VI-associated neurodegeneration (PLAN, NBIA type II; INAD1; Karak syndrome)	Globus pallidus and substantia nigra (in <50% of patients)	Infantile neuroaxonal dystrophy: hypotonia, visual disturbance, motor and mental retardation. Atypical neuroaxonal dystrophy (late onset): dystonia, dementia, and parkinsonism
Mitochondrial membrane protein-associated neurodegeneration (MPAN)	Globus pallidus and substantia nigra	Dysarthria, gait abnormalities, dystonia, and parkinsonism
Fatty acid hydroxylase-associated neurodegeneration (FAHN)	Globus pallidus and substantia nigra in some patients	Dysarthria, gait abnormalities, dystonia, and parkinsonism
COASY protein-associated neurodegeneration (CoPAN)	Globus pallidus and substantia nigra	Oro-mandibular dystonia with dysarthria and parkinsonism, cognitive impairment
β-Propeller protein-associated neurodegeneration (BPAN)	Globus pallidus and substantia nigra	Parkinsonism, dystonia, dementia, and global development delay
Kufor–Rakeb syndrome	Globus pallidus and substantia nigra in few cases	Dystonia and parkinsonism
Woodhouse–Sakati syndrome	Globus pallidus and substantia nigra in few cases	Dystonia and deafness

We are particularly interested in iron homeostasis and the mechanisms underlining its accumulation in the brain. By this point of view, the genes so far associated with different NBIA forms can be distinguished between those that code for proteins directly involved in iron metabolism and those that encode proteins responsible for other functions such as fatty acid metabolism and lysosomal activity (**Table 2**). The proposal of hypotheses about iron accumulation mechanisms may be relatively easy in the first case; while for the other type of defects it could represent a difficult challenge.

**Table 2:** Causative genes of the neurodegeneration with brain iron accumulation disorders.

Gene/disorder	Function	Cellular location	Molecular process
*Ceruloplasmin (Cp)/aceruloplasminemia*	Cu-dependent ferroxidase	Secreted – plasma membrane	Iron metabolism
*Ferritin light chain (FTL)/neuroferritinopathy*	Iron storage	Cytosol	Iron metabolism
*Pantothenate kinase-2 (PanK2)/PKAN*	Vitamin B5 phosphorylation	Mitochondria	Coenzyme A synthesis (fatty acid metabolism)
*Phospholipase A2, Group VI (PLA2G6; iPLA2β)/PLAN*	Hydrolysis of ester bonds at the sn-2 position of phospholipids	Mitochondria and cytosol	Membrane phospholipids turnover	
*C19orf12/MPAN*	Unknown	Mitochondria	Lipid metabolism?
*Fatty acid 2-hydroxylase (FA2H)/FAHN*	Synthesis of 2-hydroxysphingolipids	Endoplasmic reticulum	Myelin synthesis	
*COASY/COPAN*	CoA synthase (4′-PP adenyltransferase and dephospho-CoA kinase)	Mitochondria and cytosol	Coenzyme A synthesis (fatty acid metabolism)
*WDR45/BPAN*	Putative role in autophagy	Autophagosome?	Autophagy
*ATP13A2; PARK9/Kufor–Rakeb syndrome*	P-type ATPase – divalent cation pump	Lysosome	Lysosomal degradation – autophagy
*C2orf37/Woodhouse–Sakati syndrome*	Unknown	Nucleolus?	Unknown

Here we will try to critically resume the main results obtained from *in vitro* and *in vivo* studies of the pathogenic molecular mechanisms with the aim to highlight the iron involvement in the NBIA pathogenesis, which is still far to be clarified, while for a comprehensive review of clinical symptoms, phenotype and how to make a differential diagnosis we refer to several recent papers appeared in the literature (Kruer and Boddaert, 2012; Rouault, 2013; Schneider et al., 2013).

## NBIA CAUSED BY DEFECTS IN GENES CODING FOR PROTEINS OF IRON METABOLISM

Until now, only two genes coding for iron proteins have been identified as responsible of NBIA subtypes: the ceruloplasmin gene (*CP*) causing aceruloplasminemia (Miyajima et al., 1987) and the L-ferritin gene (*FTL*) altered in neuroferritinopathy (Curtis et al., 2001). Defects in these genes lead to early deposits of iron in the striatum, thalamus, globus pallidus (GP), dentate nuclei, cortex, retina; the clinical symptoms typically manifest in adulthood, suggesting that the brain possesses excellent compensatory mechanisms to buffer the harmful action mediated by iron. Neurologic manifestations include blepharospasm, oro-lingual-mandibular dystonia, dysarthria, chorea, parkinsonism, ataxia, and cognitive decline (Dusek and Schneider, 2012).

### ACERULOPLASMINEMIA

Aceruloplasminemia (MIM 604290) is an autosomal recessive inherited disease. It was originally described by Miyajima in a Japanese female in 1987 (Miyajima et al., 1987). Effectively, it affects particularly the Japanese population with a prevalence of approximately 1 per 2,000,000 in non-consanguineous marriages, while other 35 families have been described around the world (Kono, 2013). The symptoms include neurological signs with first appearance in adulthood (fourth or fifth decade of life), usually preceded by diabetes mellitus, retinal degeneration (Kono, 2012) and microcytic anemia, unresponsive to treatment with iron, accompanied by undetectable serum ceruloplasmin, high serum ferritin levels (from 3 to 40 times the normal levels), and low levels of sideremia (Ogimoto et al., 2011). MRI analysis of aceruloplasminemia patients reveals abnormal low intensities in the liver as well as in the striatum, thalamus, and dentate nucleus of the brain on T1 and T2 weighted images, which are consistent with iron deposition (Miyajima, 2003).

Up to now analysis of autopsies from six cases of aceruloplasminemia patients have been reported (Morita et al., 1995; Yoshida et al., 1995; Gonzalez-Cuyar et al., 2008; Kaneko et al., 2012). They showed severe destruction of the basal ganglia and dentate nucleus, with considerable iron deposition in neuronal and glial cells. In the majority of them the cerebral cortex showed mild iron deposition in glial cells without neuronal involvement, however in one case, in which the pathology lasted for the longest time, iron overload was detected also in the cerebral cortex, indicating that the neuropathologic process in aceruloplasminemia worsened during the time and extended beyond the basal ganglia to the cerebral cortex (Kaneko et al., 2012). A sustained accumulation of iron occurs also in retina and cerebellum other than in the pancreas and myocardium (Kaneko et al., 2012). Iron deposition was detected in perivascular areas, localizing to terminal astrocytic processes, especially in the basal ganglia, which show neuronal loss and accumulation of large iron-rich globular structures that appear to be the remains of dead astrocytes (Gonzalez-Cuyar et al., 2008). Accordingly, enlarged or deformed astrocytes and spheroid-like globular structures are characteristic neuropathological findings in aceruloplasminemia.

*CP* is a single-copy gene on chromosome 3, which contains 20 exons with a total length of about 65 kb (Patel et al., 2000), and encodes ceruloplasmin (Cp). The genetic analysis of patients affected by aceruloplasminemia revealed more than 40 distinct causative mutations (Kono, 2013).

Cp is a glycoprotein of the α2-globulin fraction of the serum. It is a multicopper ferroxidase, containing 95% of the copper in the plasma. Its functional role is to facilitate iron export, mediated by ferroportin, from cells. It oxidizes the Fe^2^^+^ to Fe^3^^+^ so that the ferric iron can bind to transferrin present in the extracellular environment. In central nervous system (CNS), Cp is expressed as a glycosylphosphatidylinositol (GPI)-linked form in the astrocytes (Patel et al., 2002). Its action is essential in this cerebral cell type for which the Cp is the only existing ferroxidase (Jeong and David, 2003). In the absence of Cp activity, the ferrous iron that enters the CNS cannot be oxidized and is internalized in large amount, through transferrin-independent, non-regulated pathway (Brissot et al., 2012). The excess import of iron, associated to the export inability due to ferroportin malfunctioning in the absence of Cp, leads to the remarkable accumulation of iron within astrocytes observed in the pathology. Thus it is reasonable to think that iron sequestration by astrocytes may induce iron deficiency and death in neurons, which are astrocytes-depended for iron acquisition (Jeong and David, 2006). Other cells in the CNS, including oligodendrocytes, express hephestin as alternate ferroxidase (Wang et al., 2007) and are not dependent on the action of Cp; this explains the specificity of astrocytes and neuronal death. In brain tissues and cerebral fluid there is also evidence of a marked increase in oxidative stress such as lipid peroxidation and protein carbonylation, in support to excess iron-toxicity (Kono and Miyajima, 2006).

The molecular pathogenesis of aceruloplasminemia was investigated by analysis of Cp mutants expressed in mammalian cell culture (Hellman et al., 2002; Kono et al., 2007, 2010; di Patti et al., 2009) and by characterizing murine models (Harris et al., 1999; Patel et al., 2002; Yamamoto et al., 2002).

The *in vitro* biological analysis of Cp mutants revealed three different types of pathological mechanisms, all resulting in loss of the protein ferroxidase activity. The protein structural modifications induced by mutations can lead to: (i) retention of Cp in the endoplasmic reticulum (ER), (ii) miss-incorporation of copper into apoceruloplasmin, and (iii) impaired ferroxidase activity (Hellman et al., 2002; Kono et al., 2007, 2010; di Patti et al., 2009). All these events hinder iron export from the cell, leading to cellular iron overload.

The *in vivo* experiments were performed on different mice models obtaining variable results. The first knockout mice generated on a C57BL/6J genetic background were described by Harris et al. (1999). The mice showed increased iron content with lipid peroxidation in the brain, but they did not suffer of neurological symptoms; however, a more recent work revealed that this *CP*-deficient young adult mice showed an anxiety phenotype, without discernable effects on learning and memory or motor performance (Texel et al., 2012). The authors determined that in contrast to peripheral tissues, iron levels in the hippocampus were significantly reduced in *CP*-KO mice and, paradoxically, that the anxiety phenotype resulted from reductions in the levels of iron, serotonin, and brain-derived neurotrophic factor expression in the hippocampus (Texel et al., 2012).

A second knockout mice model was developed with a different strategy by Patel et al. (2002). Also in this case *CP*-null mice showed increased iron deposition and lipid peroxidation in several regions of the CNS, but, in addition, they showed deficits in motor coordination that were associated with a loss of brainstem dopaminergic neurons. Astrocytes isolated from the CNS of these *CP*-null mice were used to investigate the functional role of Cp in CNS (Jeong and David, 2003). The authors assessed the ^59^Fe influx and efflux from astrocytes and concluded that GPI-Cp is essential for iron efflux, while is not involved in regulating iron influx. Furthermore, they identified the co-localization of GPI-Cp on the astrocyte cell surface with the divalent metal transporter IREG1 (now renamed ferroportin) and defined that the harmonized actions of GPI-Cp and IREG1 is required for iron efflux from neural cells. If disruption of this equilibrium occurs, it could lead to iron accumulation in the CNS and neurodegeneration, highlighting the importance of Cp in maintaining iron homeostasis in brain.

In 2002, Yamamoto described a third type of knockout obtained on C57BL/10 and BALB/c genetic background (Yamamoto et al., 2002). The mice had hepatic iron overload but no brain iron accumulation was detected. This murine model was then also investigated for the age dependent expression of hephestin (Cui et al., 2009). The authors detected regional difference in age-dependent hephestin expression and concluded that hephestin may compensate for the loss of Cp in a region-specific manner (Cui et al., 2009). These interesting results might explain the evidence that adult *CP*-null mice have increased iron deposition in the cerebellum and brainstem, while other regions (such as the caudate and putamen), appeared to have normal iron levels (Patel et al., 2002). Unfortunately, the latter brain regions show iron accumulation in aceruloplasminemia patients, indicating the limit of this murine model in recapitulating the aceruloplasminemia human phenotype.

The murine model that lacks the action of both Cp and hephestin develops symptoms consistent with those shown by aceruloplasminemia patients (Hahn et al., 2004; Schulz et al., 2011). Together with iron accumulation in both gray and white matter oligodendrocytes (Schulz et al., 2011) these mice also showed macular degeneration, iron overload and increased oxidative stress in the retina, (Hadziahmetovic et al., 2008). The use of a chelating agent, such as oral deferiprone (DFP), was shown to be protective against the increased oxidative stress and retinal degeneration (Hadziahmetovic et al., 2011; Wolkow et al., 2012). Despite the good results obtained in the mouse model, the use of DFP on a patient resulted in a worsening of symptoms (Mariani et al., 2004), while variable results ranging from mild amelioration to no effect on neurological symptoms were obtained in patients treated with deferasirox (Skidmore et al., 2008; Roberti Mdo et al., 2011). However iron-chelating therapy appeared effective in reducing the hepatic and pancreatic iron overload (Finkenstedt et al., 2010).

Thus, the cascade of events leading to neuronal death remains to be fully elucidated but the overall data strongly suggest that oxidative stress, driven by heavy metal accumulation, represents the primary cellular cytotoxic process, determining the neuronal damage in affected brain regions. Nevertheless, it has been described that some mutated forms of Cp can also accumulate in aggregates and lead to death of astrocytes through an iron-independent pathway (Kono et al., 2006).

### NEUROFERRITINOPATHY

The Neuroferritinopathy (OMIM, 606159, also labeled as hereditary ferritinopathies or NBIA3) is a rare monogenic autosomal-dominant progressive movement disorder caused by mutations in the gene encoding the L chain of ferritin (FtL). Ferritin (Ft) is the main protein iron storage from prokaryotes to mammals and is characterized by a highly conserved structure that consists of a virtually spherical shell with an internal cavity that can accommodate up to 4500 iron atoms. In vertebrates Ft consists of 24 subunits of two types, H and L, which are assembled in different proportions (Arosio and Levi, 2010). The three dimensional structure of the two chains is very similar: a bundle of four parallel helices, with a long loop that connects helix B and helix C, and a fifth smaller helix called E, at the C-terminus, which is directed toward the center of the cavity. The H chain has a ferroxidase center, where the oxidation of iron occur (Lawson et al., 1991), while the L chain facilitates the mineralization of the iron in the cavity supporting the ferroxidase activity of the H chain (Levi et al., 1994), thus the heteropolymer incorporates iron more efficiently than homopolymers, both *in vitro* and *in vivo* (Santambrogio et al., 1993).

**FIGURE 1:**
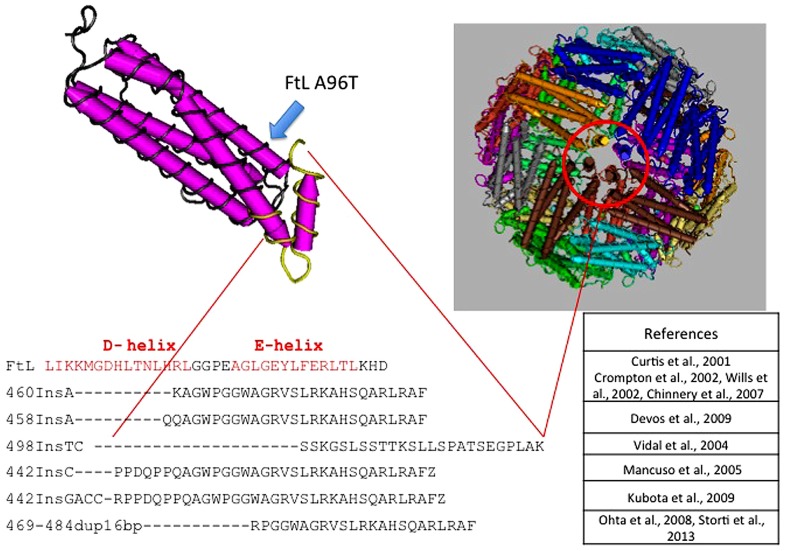
**Pile up of the C-terminus amino acid sequences of L-ferritin and the mutants causing neuroferritinopathy**. All mutations, localized in the exon 4 of FTL, are nucleotide insertions that cause large alterations of the C-terminal region of the subunit. This peptide portion forms the E-helix region, which is involved in the formation of the hydrophobic channel of the ferritin shell. Position of the A96T mutation is indicated by a blue arrow along the C-helix.

The disease was initially described in members of an English family (Curtis et al., 2001; **Table 3**), which carried the insertion of an adenine in *FTL* gene, resulting in alteration of the C-terminus of the protein encoded both in terms of sequence and of length (Curtis et al., 2001). To date, six other alterations have been identified, all of them localized in exon 4 of the *FTL* gene. All the mutations alter the helix E of the FtL and determine a change in the C-terminal part of the protein (**Figure 1**). In addition, a missense mutation, causing A96T substitution on helix C, it has been described as causative of the disorder (Maciel et al., 2005). The average age of onset is 39 years and the main clinical manifestations are those that characterize the extrapyramidal disorders. Despite all the mutations determine common manifestations such as oro-buccal dyskinesia, chorea, and dystonia, it seems that there may be subtle phenotypic variations between them in terms of age of onset, progression of the disease, or the presence of cognitive deterioration (Lehn et al., 2012).

**Table 3:** Reported cases of neuroferritinopathy.

Origin	Reference	Mutations	Symptomatology	Serum ferritin (normal range 15–250 μg/l)
Cumbrian region (UK)	Curtis et al. (2001)	460–461 dupA	Extrapyramidal dysfunction	4–16
Northwest of UK	Wills et al. (2002)	460–461 dupA	Extrapyramidal dysfunction including palatal tremor and cognitive decline	60
North of UK (10 families)	Crompton et al. (2002)	460–461 dupA	Extrapyramidal dysfunction	N.D.
France	Chinnery et al. (2007)	460–461 dupA	Dystonia, dysarthria, chorea, parkinsonism, blepharospasm, cerebellar signs, and mitochondrial respiratory chain defects	N.D.
Gypsy ancestry	Maciel et al. (2005)	474>A; A96T	Parkinsonism, ataxia, and corticospinal signs	16
France	Vidal et al. (2004)	498–499 dupTC	Tremor, cerebellar ataxia, parkinsonism and pyramidal signs, behavioral disturbances, cognitive dysfunction	N.D.
French Canadian and Dutch ancestry	Mancuso et al. (2005)	442 dupC	Dystonia, dysarthria, chorea, blepharospasm, cerebellar signs, and mitochondrial respiratory chain defects	14
France	Devos et al. (2009)	458 dupA	Dystonia, dysarthria, dysphagia	ND
Japan	Kubota et al. (2009)	442 dup4bp	Chorea, tremor, dyskinesia, dysarthria, dysphagia	46
Japan, Italy (*de novo*)	Ohta et al. (2008), Storti et al. (2013)	469–484 dup16nt	Hypotonia, hyperextensibility, aphonia, and cognitive impairment	5

In these patients, routine blood tests are usually normal, only serum ferritin may be low (**Table 3**), while electrophysiological analysis of the cerebrospinal fluid did not show appreciable differences (Lehn et al., 2012). Standard histochemical analysis of muscle biopsies are usually normal, however, Chinnery et al. (2007) found a significant percentage of cytochrome *c* oxidase-negative fibers in two of nine patients analyzed, in addition to isolated or combined defects of respiratory chain complexes in five of six patients analyzed. Iron deposits in the cerebellum, basal ganglia and motor cortex are visible by MRI, either with traditional gradient echo sequences (T2*) or with susceptibility weighted imaging (Ohta and Takiyama, 2012). The neuropathological data available were obtained from patients with c.442dupC (p.His148ProfsX33; Mancuso et al., 2005), the c.460dupA (p.Arg154LysfsX27; Curtis et al., 2001) and c.498dupTC (p.Phe167SerfsX26; Vidal et al., 2004). The examination of the brains of these patients showed mild cerebral and cerebellar atrophy as well as cavitation of the putamen. The main neuropathological findings were the presence of intracytoplasmic and intranuclear aggregates of Ft in the glial cells and in some neuronal subtypes, deposits of iron, gliosis, and neuronal death. Glial cells that contain aggregates were found mainly in the caudate, putamen, and GP, and these areas also showed the death of nerve cells and extracellular deposits of Ft. In the cerebral cortex, aggregates were found in cells of the perineural and perivascular glia. The presence of aggregates in neurons was clearly visible in the putamen, the GP, thalamus, in cerebellar granule, and Purkinje cells (Curtis et al., 2001; Vidal et al., 2004; Mancuso et al., 2005).

The aggregates appeared as homogeneous eosinophilic bodies, that were stained with antibodies anti-FtL, anti-FtH or against the mutated form of the *FTL*; inclusions contained both Fe^2^^+^ and Fe^3^^+^, the presence of which was analyzed respectively using Turnbull blue and Perls’ Prussian blue. Using the transmission electron microscopy, nuclear aggregates appeared as granules of about 100 Å, reminiscent of the structure of Ft and occupy a large part of the nucleoplasm. The presence of aggregates was also reported in cells of other tissues such as skin, liver, kidney, and muscle (Vidal et al., 2004; Mancuso et al., 2005).

Biochemical analysis of the isolated aggregates identified FtH, FtL, and FtL variant as components, suggesting that the variant form assembles in the ferritin heteropolymers (Vidal et al., 2004). Extensive work has been done on variant recombinant proteins with the attempt to define the structural alteration induced by mutations (Baraibar et al., 2008, 2010; Luscieti et al., 2010). The crystallization of the p.Phe167SerfsX26 homopolymer (Baraibar et al., 2008, 2010; Luscieti et al., 2010) showed that the mutated L chain overlaps exactly with the wild type chainup to glycine 157 while the downstream portion of the amino acids sequence is not solvable, suggesting that the final part of the chain is unstructured (Baraibar et al., 2008, 2010; Luscieti et al., 2010). This alteration of the C-terminal and the exposure of this part of the chain determines a disorganization of the channel described above with two main consequences: a reduced physical stability of the protein due to the loss of stabilizing interactions along the axis of symmetry and the formation of a wider and permeable quaternary channel. These alterations of the hydrophobic channel in the heteropolymer occur also when only one of four subunits, that make up the channel, is changed, thus indicating a dominant negative effect exerted by the mutant protein (Luscieti et al., 2010). Muhoberac et al. (2011) confirmed a greater propensity of ferritin to precipitation induced by iron and a lower functionality of the heteropolymer containing variant chains. Recently, it has also been reported a greater propensity to oxidation of the mutated chains, both *in vitro* and *in vivo*, stressing that oxidative stress is a key component of the pathogenesis (Baraibar et al., 2012).

**FIGURE 2:**
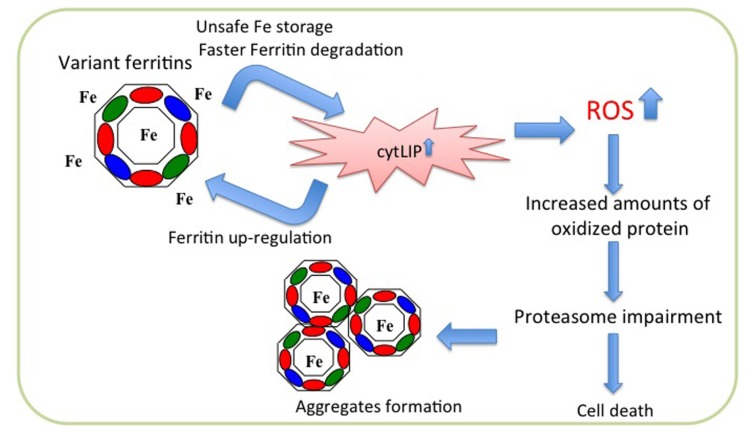
**Scheme of pathogenetic molecular mechanism of neuroferritinopathy**. The mutated peptide (green ellipse) assembles with the H- (red ellipse) and L- (blue ellipse) subunits to form ferritin shell, which is unable to incorporate iron properly. This leads to iron excess in the cytosol (cytLIP), which induces iron-dependent ferritin translation, generating a self-maintained vicious cycle, and at the same time stimulating ROS production and oxidative damage. In long period this causes impairment of the proteasome, ferritin aggregation, and cell death.

Overexpression of neuroferritinopathy variants in cells led to the proposal of a hypothetical pathogenetic pathway (Cozzi et al., 2006, 2010). Stable transfection of the p.Arg154LysfsX27 in HeLa cells revealed that the mutant chain assembles in the Ft heteropolymers, although in low proportion (on average less than four subunits for polymer); its expression is associated with an increase in the endogenous ferritin chains expression and a decrease in the expression of transferrin receptor 1 (TfR1). In addition, an increase of reactive oxygen species (ROS) production is evident after treatment with H_2_O_2_ in comparison to control cells. The polymer containing the mutated chains incorporates iron with lower efficiency and is degraded much faster than the wild type protein. This leads to an increased release of iron in the cytoplasm, which stimulates the expression of the endogenous ferritin chains, that assemble with the mutant one thus establishing a vicious cycle (Cozzi et al., 2006; **Figure 2**). Similar data were obtained also with expression of p.Phe167SerfsX26 in neuroblastoma cell lines (Cozzi et al., 2010). When compared to control cells, both variant lines showed an increase of endogenous ferritins, an increase in the LIP after treatment with iron and an enhanced production of ROS after treatment with H_2_O_2_, accompanied by an intensification in protein ubiquitination. Both cell lines develop aggregates positive for FtL and iron, which grow in number and size upon iron supplementation, and a defect in the proteasomal activity, which is rescued by treatment with iron chelator and antioxidant agents (Cozzi et al., 2010). These data are confirmed by the analysis of fibroblasts from a patient carrying the c.498dupTC mutation (Barbeito et al., 2010). These cells show an altered management of iron, an accumulation of ferritin and markers of oxidative stress (Barbeito et al., 2010). In basal conditions, the patient’s fibroblasts show a significant increase compared with control fibroblasts in the amount of total iron, while the LIP is not altered; the expression of H-, L-, and mutated-L Ft is increased, while that of TfR1 is decreased. Furthermore, also the binding capacity of IRP to IRE is decreased. The whole phenotype is consistent with an increase in the intracellular free iron. Even in these cells the level of ROS is significantly increased compared to that of controls (Barbeito et al., 2010).

*In vivo* data were obtained on a mouse model expressing the human cDNA of *FTL* with the mutation c.498dupTC. The expression of the transgene in the animal caused the formation of nuclear and cytoplasmic aggregates of ferritin throughout the CNS and in other organs (Vidal et al., 2008). The size and the number of nuclear aggregates increase with the aging of the animal, as is the case for patients (Vidal et al., 2004). The model shows a progressive neurological phenotype, a decreased mobility and a reduced life expectancy as well as an increase in the amount of iron in the brain with altered levels of the associated proteins. In fact, in brain the expression of H- and L-Ft is increased while TfR1 expression is decreased. The transgenic mice also show an accumulation of oxidized DNA in the mitochondria of the brain but no significant damage to the nuclear DNA (Deng et al., 2010) even if oxidative stress markers such as protein carbonylation and lipid peroxidation are elevated (Barbeito et al., 2009).

In conclusion, the overall data obtained by *in vitro* analysis of protein suggest that the pathogenesis could be caused by a reduction in ferritin iron storage capacity and by enhanced toxicity associated with iron-induced ferritin aggregates, whereas data on cellular models, confirmed by the study on transgenic mouse model, imply that the pathogenesis could be mostly related to iron-dependent oxidative damage. Thus, time should be invested now in the development of therapeutic agents aimed at blocking the detrimental cascade of oxidative events. Some promising results have been recently obtained in more common diseases, such as Parkinson’s disease (Devos et al., 2014), and might be extended to these cases.

## NBIA CAUSED BY DEFECTS IN GENES CODING FOR PROTEINS INVOLVED IN LIPID METABOLISM AND MEMBRANE HOMEOSTASIS

### PANTOTHENATE KINASE-ASSOCIATED NEURODEGENERATION

Pantothenate kinase-associated neurodegeneration (PKAN or NBIA type I) is the most frequent syndrome among NBIA disorders and it was the first one to be associated to mutations in a specific gene, namely pantothenate kinase 2 (*PanK2*; Zhou et al., 2001). It accounts for about 50% of all NBIA cases and may present in two distinct and age-dependent forms, the classic one with early onset and the atypical one, with later onset of the disease. The classic form usually manifests in the first decade of life, more often before the age of 6 years (Hayflick et al., 2003). Gait and postural difficulties are the most common symptoms often associated to extrapyramidal features such as dystonia, dysarthria, and choreoathetosis. Spasticity and hyperreflexia as well as retinopathy are often present. The atypical form most commonly presents in the second decade of life and its clinical features are less homogeneous, with less severe extrapyramidal and pyramidal signs. Difficulties with speech and psychiatric symptoms with cognitive decline are often observed.

The MRI analysis is a fundamental step in the diagnostic process given that the vast majority of the patients show a specific pattern defined as “eye of the tiger” at T2-weighted MR images. It corresponds to bilateral areas of hypointensity in the medial GP with central spots of hyperintensity (Hayflick et al., 2003). The area of reduced signal is due to iron accumulation in the tissue, as confirmed by the histology (Kruer et al., 2011), whereas the central hyperintense signal may be linked to the dramatic rarefaction of the tissue and the microglial activation (Sethi et al., 1988; Kruer et al., 2011). On the basis of MR data the iron content in the GP of PKAN patients would be about twice as much that found in controls (391 vs 177 μg/ml; Dezortova et al., 2012); the larger proportion of it would be inside ferritin as antiferromagnetic crystals, but a minor part would have different, not yet determined characteristics and have ferrimagnetic properties.

According to a recent study performed on six genetically confirmed PKAN cases (Kruer et al., 2011), the pathology of the disease was almost exclusively located in the CNS, and particularly in the GP where a strong reduction of neurons and synapsis was evident. This was accompanied by the presence of reactive astrocytes, of numerous large and granular spheroids, corresponding to degenerating neurons, and of small and more eosinophil spheroids corresponding to swollen dystrophic axons. Iron accumulation was evident in the GP already on hematoxylin–eosin stain, particularly in the form of perivascular hemosiderin deposits, sometimes within macrophages. The Perl’s staining showed the presence of iron within the GP of PKAN brain with an intensity significantly higher than that observed in control brain; the accumulated metal was particularly evident in the cytoplasm of astrocytes, but could also be detected in some neurons, and with a more diffuse pattern in the neuropil. Ferritin staining partially overlapped with the Perl’s result and was significantly increased in astrocytes, with a granular pattern, and in the neuropil; in some degenerating neurons the iron staining seemed to be more intense than that of the storage protein, ferritin. This could be in line with the suggestion of two types of iron in the GP of patients proposed from the interpretation of MR data (Dezortova et al., 2012). Overall there was no evident correlation between the intensity of iron accumulation and other histological or clinical features.

An interesting observation was the positive staining for ubiquitin found both in many degenerating neurons and in residual intact neurons present in the GP, possibly indicating the accumulation of ubiquitinated proteins as an early event in the degenerative process. Less consistent was the staining for tau or amyloid precursor protein (APP) in these cells. The latter was instead particularly evident in neuroaxonal spheroids. In contrast with previous studies performed on Hallervorden–Spatz cases without a genetic confirmation of the disease (Galvin et al., 2000; Neumann et al., 2000; Wakabayashi et al., 2000), no Lewy bodies were detected, thus challenging the interpretation of PKAN as a synucleinopathy.

Pantothenate kinase-associated neurodegeneration is an autosomal recessive syndrome (OMIM number 234200) associated to mutation in the *PanK2* gene. Mutations are usually missense, with c.1561G > A (p.G521R) and c.1583C > T (p.T528M) being the most common, but deletions, duplications, and exon splice site variations have been identified (Hartig et al., 2006). The predicted functional severity of the mutations seems to correlate with the age of onset, but not with the loss of ambulation and the course of the disease (Hartig et al., 2006).

Four *PanK* genes are present in the human genome, which code for PanK enzymes. The different isoforms (PanK1a and b, Pank2, Pank3, and Pank4) share a common C-terminal catalytic domain of about 350 amino acids with about 80% identity and differ for the N-terminal portion of the protein, which contributes to determine their specific cellular localization and regulatory properties (Leonardi et al., 2005; Alfonso-Pecchio et al., 2012; Garcia et al., 2012). PanK2 gene consists of six core exons and different initiating exons; the transcript variant 1 (NM_153638) is the most common (Polster et al., 2010) and code for a protein of 570 amino acids, with a long N-terminal domain. Targeting signals within the N-terminal part of the protein drive it to mitochondria, where two sequential proteolytic cleavages by the mitochondrial processing peptidase lead to the mature, long-lived and active protein, with a molecular mass of 48 kD (Kotzbauer et al., 2005). The alternate use of a downstream CTG codon for translation initiation was described, that would give rise to a protein of 50.6 kD, always with a mitochondrial localization (Zhou et al., 2001; Johnson et al., 2004). Other transcript variants are described, but they are predicted to code short, not functional cytosolic proteins. Different approaches have shown that the mature PanK2 protein resides within the mitochondrial intermembrane space (Alfonso-Pecchio et al., 2012; Brunetti et al., 2012). Interestingly, a recent analysis of PanK proteins cellular compartmentalization (Alfonso-Pecchio et al., 2012) confirmed a preliminary observation by Hörtnagel et al. (2003) and showed a nuclear localization for PanK2, with the identification of nuclear targeting and export signals driving the entry of the protein into the nucleus and the subsequent exit for the final trafficking to the mitochondria. The functional protein exists as a dimer. The mouse *PanK2* gene structure is the same of the human gene, yet there are conflicting reports as to the cellular localization of the murine protein, alternatively documented in the cytosol (Leonardi et al., 2007b; Alfonso-Pecchio et al., 2012) or in the mitochondria (Brunetti et al., 2012).

A full comprehension of the mechanism linking defects in PanK2 functioning with the neurodegenerative process and iron accumulation in the brain is lacking yet, even though very recent data provided new perspectives, also by the therapeutic point of view. To define the biological mechanism underpinning the development of this disorder different aspects await for clarification. Is the perturbation of CoA biosynthesis the main pathogenic trigger or does PanK2 have other biological functions? What are the relevance and the specific role of Pank2 in CoA biosynthesis and what is the nature of its relationship with the other isoforms of the enzyme? What is the connection linking Pank2 activity and iron metabolism? Why is the brain and particularly specific cerebral areas and neurons so vulnerable to the degenerative process? Even though many of the previous questions remain unanswered, much progress has been recently obtained by different approaches ranging from biochemical *in vitro* studies to the use of cellular and animal models of the disease.

**FIGURE 3:**
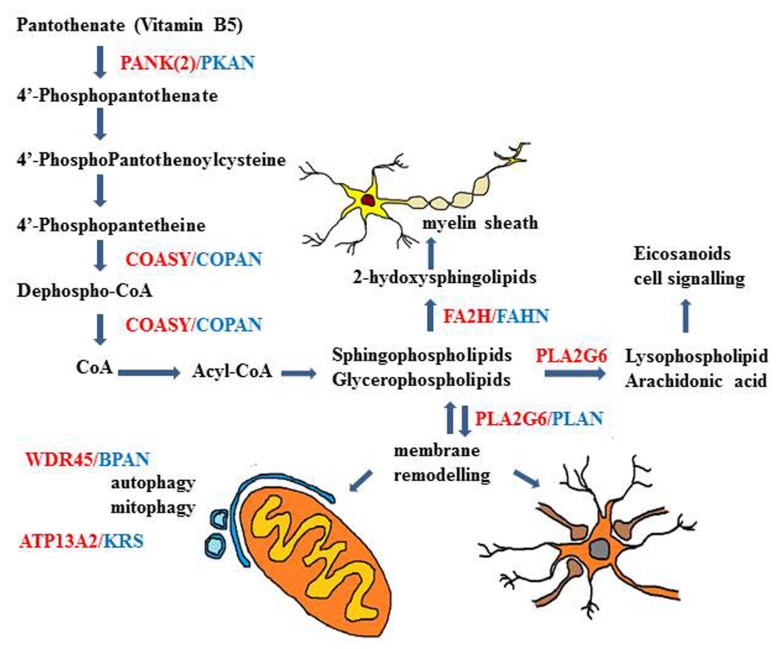
**Schematic description of genes and biochemical pathways involved in different types of NBIA disorders**. The biochemical pathways of lipid metabolism and membrane/organelles (mitochondria) remodeling seem to play an important mechanistic role in many of the genetic NBIA disorders so far identified.

The recognized function of Pank2 is to catalyze the first and limiting step of CoA biosynthesis, that is the phosphorylation of pantothenate (vitamin B5) to 4′-phosphopantothenate (**Figure 3**). This was shown *in vitro*, in transfected cells, where the increased catalytic activity was predominantly found in mitochondria (Kotzbauer et al., 2005; Zhang et al., 2006b). The enzymatic activity was inhibited by CoA (IC_50_ = 50 μM) and more strongly by a CoA ester (IC_50_ = 1 μM). This block could be overcome by palmitoylcarnitine, that works as a potent activator of PanK2 function (Leonardi et al., 2007a). This explain how Pank2 can function *in vivo* with the physiological concentration of CoA and acyl-CoA esters and suggests that the presence of enzyme within the mitochondria inner space could be important to sense the levels of palmitoylcarnitine and modulate CoA biosynthesis according the requirement for β-oxidation. Interestingly the *in vitro* analysis of different PanK2 mutants showed that in many cases the cellular localization, the enzymatic activity, and the regulatory properties are all maintained or only modestly affected (Kotzbauer et al., 2005; Zhang et al., 2006a; Leonardi et al., 2007a). This is for instance the case with the c.1583C > T (T528M) mutation that occurs with high frequency in both early and late onset diseases. Experiments performed with wild type and mutant hPank2 in the fumble (*fbl*) fly model (Wu et al., 2009b) partially questioned the reliability of these *in vitro* enzymatic assays and showed a better correlation between loss of Pank2 catalytic activity and rescue potency. Nonetheless the alteration of PanK2 catalytic activity may not be the only cause of the neurodegenerative process and the possibility of other yet unknown functions of the protein has to be considered. As a matter of fact no definitive evidence is available showing decreased level of CoA in human Pank2 deficient cells or tissues. Recently, the CoA levels in mice with the genetic deletion of *Pank2* alone or in combination with *Pank1* (dKO) were analyzed; no decrease was detected in any tissue in adult *Pank2*-KO mice, while a significant reduction was found in the brain of dKO pups (Garcia et al., 2012). The study suggests that the expression of the other PanK isoforms as well as that of CoA-degrading enzyme such as Nudt7 and Nudt19 may compensate for the absence of Pank2 function. Whether this occurs also in the human brain is not known.

Even though the precise role of Pank2 in CoA biosynthesis in not fully understood yet, the strong connection between this metabolic pathway and neurodegenerative processes is confirmed by the recent identification of mutations in *COASY* in two subjects with clinical and MRI features of NBIA (Dusi et al., 2014). COASY is a bifunctional enzyme with 4′-PP adenyltransferase (PPAT) and dephospho-CoA kinase (DPCK) activities, catalyzing the last two steps in the CoA biosynthesis. Interestingly, in contrast to previous reports showing localization of COASY at the outer mitochondrial membrane (Tahiliani and Neely, 1987; Zhyvoloup et al., 2002), these authors found the enzyme in the mitochondrial matrix, probably anchored to the inner membrane. In the light of this finding the possibility of a CoA biosynthetic process inside the mitochondria has to be further considered and may explain the specificity of Pank2 in this metabolic pathway.

Relevant insight into the biological mechanism connecting defects of Pank2 function, CoA metabolism and the neurodegenerative process came from the drosophila model of the disease. *Drosophila* has only one PanK gene, *fbl*. It gives rise to different transcripts; the longest one (Fbl/L) seems to code for a protein of 512 amino acids with a mitochondrial localization, while the other proteins are cytosolic (Wu et al., 2009b). *Fbl1*, a hypomorphic allele, is due to a P-element insertion near the fbl locus. Fbl1 flies show defects in mitosis, male and female sterility, locomotor dysfunction, neurodegeneration, and reduced life-span (Afshar et al., 2001; Bosveld et al., 2008). The levels of CoA in the mutant flies are significantly lower than in control animals and they are also reduced in *Drosophila* S2 cells exposed to specific fbl silencing (Rana et al., 2010); in deficient animals and cells, mitochondria are severely affected, with damaged and ruptured cristae and membranes, and oxidized proteins are significantly increased. The reduction of *de novo* CoA synthesis is also associated with a marked decrease in the acetylation of histones and tubulin (Siudeja et al., 2011), proteins that are involved in neurodegenerative processes (Fischer et al., 2010). The restoration of appropriate acetylation levels by use of HDAC inhibitors partially corrected many aspects of the phenotype of *Pank2*/*fbl*-deficient cells, such as radiation sensitivity, climbing activity, and homozygous exclusion rate. Furthermore, reduced CoA levels led to hyperphosphorylation and inhibition of twinstar, the fly homolog of cofilin, an essential factor for actin remodeling (Siudeja et al., 2012). These biochemical changes are mediated by pathways that involve Cdi kinase and slingshot phosphatase activities and led to morphological changes in cells and compromised the differentiation and neurite formation capability of human neuroblastoma cells exposed to retinoic acid. Of extreme relevance the fact that the addiction of pantethine to the diet of the mutant flies or to the culture medium of *Pank2/fbl*-deficient cells determined the correction of many of the described biochemical and functional features (Rana et al., 2010; Siudeja et al., 2011, 2012). The metabolite is probably reduced to pantetheine, and then converted to 4’-phosphopantetheine, an intermediate of the canonical *de novo* biosynthesis pathway downstream of PanK2 function. This piece of evidence reinforces and strengthen the direct connection between CoA metabolism and the neurodegenerative process of PKAN. Very recently an alternate drosophila model has been generated with exclusive suppression of *fbl* in tissues with circadian clock cells, that is eyes, fat and specific neural cells (Pandey et al., 2013). The phenotype of these flies recapitulated aspects observed in the *flb1* hypomorphic animals, with increased sensitivity to oxidative stress, developmental lethality, and shorter lifespan; the transcriptome analysis revealed features partially overlapping with those observed in flies exposed to paraquat, that is in pro-oxidant conditions, but also a specific signature with perturbed expression of genes involved in mitochondrial pathways, cytoskeleton assembly, cell surface receptor signaling, and eye pigment biosynthesis, that could be particularly relevant in the development of retinal degeneration. Altogether the analysis confirmed the relevance of mitochondria and oxidative stress in the pathogenesis of the disease, but also indicated a wide range of transcriptional effects induced by Pank2/Fbl defects and CoA deficiency, that could explain phenotypes observed in different models, such as those related to cytoskeleton function and protein acetylation (Siudeja et al., 2011, 2012) and to iron metabolism (Poli et al., 2010).

The analysis of the mouse model of the disease was at first disappointing, but more recently it has provided new important insight into the disease pathogenesis and possibly opened new therapeutic perspectives. *Pank2* null mice were obtained by Kuo et al. (2005) and were followed for more than 1 year. When compared to littermates they showed a 20% reduction in size and were infertile because of a block in spermiogenesis, with absence of elongated and mature spermatids in the testis. Over time they showed a progressive retinal impairment with alteration of the retinal layers with cones and rods. No sign of motor dysfunction nor of iron deposition in the brain were evident, as assessed either by MRI and by histochemistry, even after 16 months of age or after backcrossing into the C57/BL6J strain. With the exception of retinal degeneration, these features were confirmed in another knock out mouse (Garcia et al., 2012). Tissues, form the latter model, were also investigated for CoA level, and no difference was observed in comparison to age-match littermates. Interestingly, these authors reported that the deletion of *Pank2* resulted to be lethal in the C57/BL6J strain. Brunetti et al. (2012, 2014) further analyzed the *Pank2*^-/-^ mice and found a profound alteration of the mitochondria, both in cultured neurons and in the CNS and peripheral nervous system (PNS); these organelles were swollen with altered cristae and membrane potential and oxygen consumption was significantly reduced, albeit the function of respiratory chain complexes appeared to be normal. This was in perfected agreement with the observation of damaged mitochondria in the *fbl* hypomorph fly. When *Pank2*^-/-^ mice were fed with a ketogenic diet for 2 months, they developed clear signs of motor and neurological impairment, with foot clasping, tail rigidity, and dystonic limb positioning. The histology revealed the presence of cytoplasmic, eosinophilic, PAS-positive inclusions in some neurons. The anti-ubiquitin staining decorated these inclusions but also smaller granules in apparently intact neurons and larger ones in degenerating neural cells. The electron microscopy analysis confirmed the presence of altered mitochondria in the basal ganglia of *Pank2*^-/-^ mice; the phenomenon was dramatically worsened by exposure to the ketogenic diet. Damaged mitochondria were also found in the murine muscle and in the same tissue from a PKAN patient. All of these features were rescued when pantethine was added to the diet, thus confirming the beneficial effect of the metabolite observed in the *Drosophila* PKAN model and indicating a possible therapeutic approach for PKAN patients.

**FIGURE 4:**
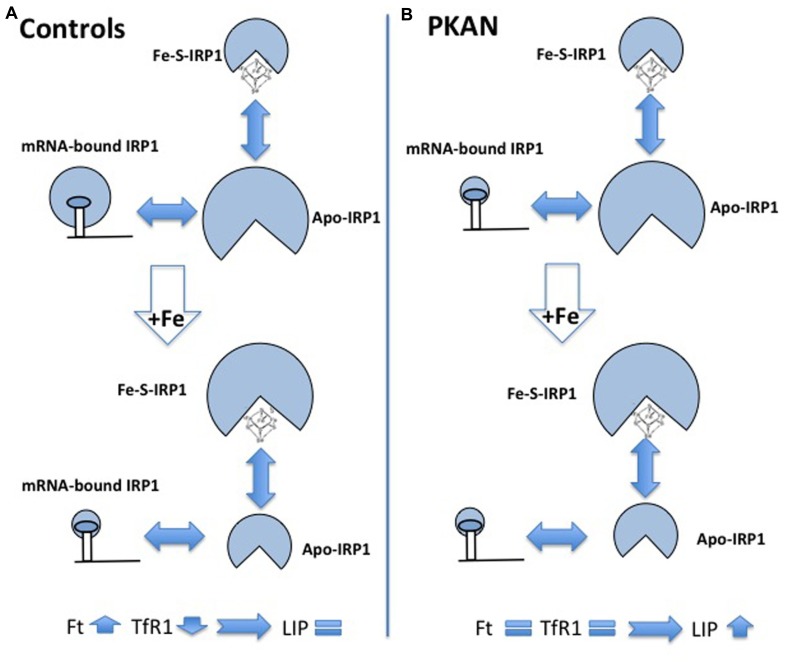
**Schematic representation of alteration of iron homeostasis control in PKAN fibroblasts**. The scheme shows the different structural conformations of IRP1 (Apo, Fe–S, and mRNA-bound) in basal condition and after iron addition, in control **(A)** and in PKAN **(B)** cells. In basal condition, the amount of the regulatory functional form (mRNA-bound IRP1) is lower in PKAN than in controls fibroblasts. Controls cells respond to iron addition, reducing the amount of mRNA-bound IRP1 and leading to up-regulation of ferritin (Ft) and down-regulation of transferrin receptor 1 (TfR1). This does not occur in PKAN cells where the levels of Ft and TfR1 do not change allowing free iron increase.

The accumulation of iron in the GP is a constant feature of PKAN patients but no explanation of the phenomenon is available and very little has been done to understand it, also because the available animal models of the disease showed no sign of iron accumulation. A possible interpretation of the phenomenon was linked to the detection of increased glutathione–cysteine mixed disulfide together with reduced cysteine deoxygenase activity in the GP of PKAN patients (Perry et al., 1985). The accumulated cysteine would chelate iron and induce an abnormal production of ROS with consequent damage to lipids and neuronal membranes. Two works have studied cellular models to investigate possible biochemical mechanisms linking CoA and iron metabolism. Specific siRNA silencing of *Pank2* in different human cell lines induced a mark reduction in cell proliferation together with unexpected signs of iron deficiency, with decrease of Ft and increase of TfR1 and free protoporphyrin levels (Poli et al., 2010). The amount of aconitase, an iron-dependent enzyme, was also reduced, both in the cytosol and in the mitochondria. Interestingly, the reduced level of Pank2 was associated to a remarkable increase of ferroportin expression, the sole cellular iron exporter, thus suggesting a possible linkage between Pank2 function and iron transport to the brain. The oxidative status and the response to iron supplementation were analyzed in fibroblasts from three PKAN patients and controls (Campanella et al., 2012). Sign of oxidative stress were detected in cells from patients already in basal conditions, and ROS production was increased in these cells after exposure to iron. Under these conditions the fibroblasts from PKAN patients were not able to modulate the binding of IRP1 to mRNA, which in turn resulted in defect in the regulation of ferritin and TfR1 and in higher amount of intracellular free iron (LIP; **Figure 4**). The data suggest that the increased production of ROS associated to Pank2 defects can perturb IRP1 activity and the control of LIP, thus generating the condition for further ROS production in a vicious cycle that could be extremely detrimental for neurons. Even though the biological and clinical meaning of iron accumulation in the brain of patients remains elusive, the administration of DFP, an iron chelating agent had beneficial effects and apparently reduced brain iron levels in two cases of idiopathic, adult-onset NBIA (Forni et al., 2008; Kwiatkowski et al., 2012). More recently two clinical trials have tested the therapeutic potential of DFP; while the reduction of iron in the GP was consistent, the clinical benefit was absent in one case (Zorzi et al., 2011) and only partial in the other one (Abbruzzese et al., 2011). A study on a larger cohort of patients and for longer time is undergoing (http://clinicaltrials.gov/ct2/show/study/NCT01741532).

### PHOSPHOLIPASE 2, GROUP VI-ASSOCIATED NEURODEGENERATION

*PLA2G6*-associated neurodegeneration (PLAN) is the second core NBIA syndrome (NBIA type II, OMIM 256600 and 610217) and is associated to mutations in the *PLA2G6* gene (Morgan et al., 2006). It can have two different age-related presentations, infantile neuroaxonal dystrophy (classic INAD) and atypical neuroaxonal dystrophy (atypical NAD). The former has an early onset presentation, usually with psychomotor regression and within 3 years of age and has a rapid progression, with neurological deterioration leading to loss of ambulation, four limb spasticity, truncal hypotonia, cerebellar ataxia, optic nerve atrophy. The atypical form has a later onset and a less homogeneous presentation with gait problems, ataxia or speech difficulties being the most common symptoms (Gregory et al., 2008). Dystonia and dysarthria together with neuropsychiatric features and visual defects characterize the progression of the disease that usually allows a longer life span than INAD. Rare forms of *PLA2G6*-associated dystonia-parkinsonism have been also identified, characterized by sub acute onset in early adulthood, frequently with neuropsychiatric changes and gait disturbance. The patients then develop dystonia and parkinsonism that may be accompanied by rapid cognitive decline.

In INAD patients the MRI consistently show cerebellar cortical atrophy often associated with signs of gliosis (Farina et al., 1999; Kurian et al., 2008; McNeill et al., 2008). The accumulation of iron is not a constant feature in patients. The metal deposition can be detected in the GP, substantia nigra, and dentate nuclei and usually increases with disease progression. Changes in the posterior corpus callosum and in the cerebral white matter are present. Prominent brain iron accumulation eventually with cerebellar atrophy can be the main neuroradiological sign in atypical INAD whereas non-specific changes such as cerebral atrophy are reported for individuals with *PLA2G6*-related dystonia-parkinsonism (Kurian and Hayflick, 2013).

In *PLA2G6*-associated neurodegeneration pathological changes are widely distributed throughout the CNS. Cerebral atrophy and sclerosis, with loss of neurons and gliosis, accumulation of lipid and degeneration of the optic pathway are evident (Cowen and Olmstead, 1963; Gregory et al., 2008). Depletion of cerebellar granular and Purkinje cells can be present. Numerous axonal swelling and spheroid bodies containing complex network of tubulovesicular membranes are usually detected in the CNS (Itoh et al., 1993; Gregory et al., 2008; Paisán-Ruiz et al., 2012). They are 30–100 μm in size and can be often stained with antineurofilament antibodies. Less consistent is the same observation in biopsies form skin, peripheral nerves, muscle and conjunctiva of INAD patients (Kurian et al., 2008). The analyses of brains form individuals with genetically proven PLAN (Gregory et al., 2008; Paisán-Ruiz et al., 2012) documented the presence of diffuse synuclein-positive Lewy pathology, particularly evident in the neocortex. In some cases there was evidence of tau pathology, either as threads in the dendrites and axons or as neurofibrillary tangles in neural bodies. This suggests a close relationship between PLAN and Parkinson’s disease.

Phospholipase 2, group VI-associated neurodegeneration is an autosomal recessive disease linked to mutations in *PLA2G6* gene (Morgan et al., 2006). To date different types of sequence variations have been documented, including non-sense and missense mutations, small exons deletions, splice-site, and copy number variations (Morgan et al., 2006; Wu et al., 2009a; Crompton et al., 2010). Even though no clear genotype/phenotype correlation exists, the early onset and more aggressive forms are associated with two null mutations, while the atypical presentation is more common in individuals carrying missense mutations. The dystonia-parkinsonism type is associated with mutation not affecting the catalytic domain of the enzyme (Engel et al., 2010).

**FIGURE 5:**
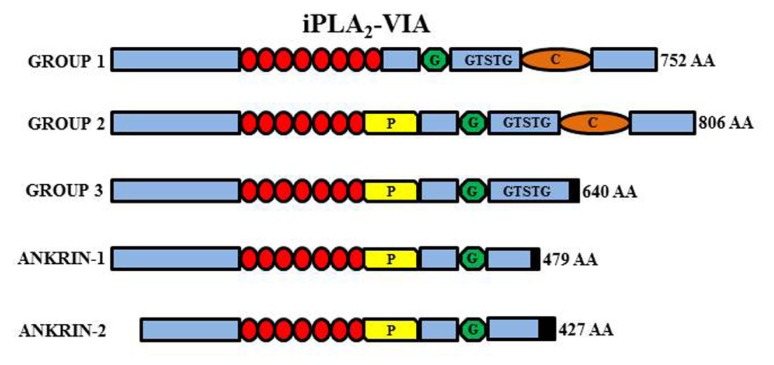
**Schematic representation of the different iPLA2-VIA isoforms**. Group VI-1 is characterized by the presence of eight ankyrin repeats (red circles), a glycine-rich, nucleotide-binding motif (G), a consensus lipase motif (GXSXG), and a calmodulin binding motif (C). Group VI-2 has the insertion of a proline-rich motif (P) in place of the eighth ankyrin repeat. Group VI-3 has a truncated COOH-terminus but conserve the lipase motif while the ankyrin-1 and -2 lack the active site and are non-functional.

The *PLA2G6* gene maps to chromosome 22q13.1 and contains 19 exons. Multiple transcript variants probably results from alternative splicing and are expressed at variable levels in most tissues. The two major isoforms (iPLA2β/iPLA2-VIA-1 and iPLA2γ/iPLA2-VIA-2) are quite similar and differ because of a lower number of ankyrin domains and a prolin-rich insertion at the N-terminus of the type 2 protein. They are both catalytically active and have a nucleotide binding-domain, a classical GSXSG lipase consensus sequence and a calmodulin-binding site at the C-terminus (**Figure 5**). The shorter isoforms (Ank-1 and Ank-2) lack the catalytic domain and the enzymatic activity. The active enzyme is a tetramer (Ackermann et al., 1994), probably enabled by interaction among ankyrin repeats. It is thus possible that the shorter inactive forms may act as dominant negative regulators/inhibitors as shown by co-transfection experiments (Larsson et al., 1998). The gene is ubiquitously expressed but seems to have a particularly relevant role in the CNS. It can be found in all regions of the mammalian brain and its activity is the most prominent among PLA2s in the rat brain (Yang et al., 1999; Balboa et al., 2002). The protein is usually considered to reside in the cytosol, but translocation to membrane compartments has been documented as well as localization in the mitochondria (Williams and Gottlieb, 2002; Seleznev et al., 2006); the latter would be an important feature shared by different proteins coded by genes associated to forms of NBIA and would point to mitochondria as central actors in the development of such disorders. iPLA2β catalyzes the hydrolysis of ester bonds at the sn-2 position of glycerophospholipids, thus releasing free fatty acids and lysophospholipids (Balsinde and Dennis, 1997; Tang et al., 1997; Burke and Dennis, 2009; **Figure 5**); the thioester bond in acyl-CoAs is also a substrate of the enzyme that, in contrast to many other members of the PLA2 family, is active in the absence of calcium. The best recognized function of the enzyme is the homeostatic regulation of membranes topology, by modulating fatty acids recycling and phospholipids amount in the membranes (Balsinde and Dennis, 1997; Winstead et al., 2000); more recently the protein has been implicated in a variety of other cellular processes associated to the generation of arachidonic acid (AA; Green et al., 2008) a precursor of eicosanoids such as prostaglandins and leukotrienes, and lipid second messengers; the involvement in cell signaling processes is well documented by the role played in the control of cells proliferation (Balboa et al., 2008; Hooks and Cummings, 2008), apoptosis (Shinzawa and Tsujimoto, 2003; Pérez et al., 2006), insulin secretion (Zhang et al., 2006a), and store regulated-calcium influx (Smani et al., 2004; Strokin et al., 2012).

The direct connection between defects in iPLA2β and the development of the neurodegenerative process is not understood yet. On the basis of the relevant role of the enzyme in controlling membrane phospholipid turnover and mass it is possible to speculate that perturbation of membrane lipid homeostasis may lead to the structure abnormalities and the axonal pathology that characterizes the disease. This is probably an important but partial aspect in the pathogenesis and further studies are needed to understand the relevance of phospholipid metabolism and iPLA2 functioning in the brain and their involvement in PLAN and other neurodegenerative disease. In contrast to PKAN, mammalian animal disease modeling has been successful for PLAN and important advancement should come from the analysis of the existing mouse models. *PLA2G6* knock out mice are less fertile because of spermatozoa malfunctioning and show defects in pancreatic β-islet activity (Bao et al., 2004; Shinzawa et al., 2008). More importantly, they developed a progressive neurologic phenotype, with gait disturbances, defects in balance and climbing, poor performance at the rotarod and the hanging grip test (Malik et al., 2008; Shinzawa et al., 2008). The motor impairment was evident at about 1 year of age, worsened with age and was associated with a shorter lifespan (Shinzawa et al., 2008).The neuropathological assessment revealed features strikingly similar to those documented in INAD patients with numerous spheroids and vacuoles affecting axons and the neuropil throughout the brain and the PNS (sciatic nerve, mesenteric and celiac ganglia); these structures were often highlighted by the staining with anti-ubiquitin antibody and sometimes were also positive for α-synuclein; they often contained PAS-positive granules. Neither Lewy bodies nor iron accumulation were evident in the brain of *PLAG26*^-/-^ mice. The ultrastructural examination evidenced a frequent presence of tubulovesicular structures, vacuoles, dense granules, and amorphous material in the spheroids. A very similar phenotype was also observed other mouse models, either expressing a non-functional protein with an amino acid substitution in the ankyrin repeat domain (Wada et al., 2009) or expressing very low level of *PLA2G6* transcript because of a viral insertion upstream of the start codon (Strokin et al., 2012). In these mice the motor impairment was already evident at 2 months of age and progressively worsened. The mice died between 18 and 24 weeks of age. The pathology was characterized by the presence of numerous spheroids, very similar to those observed in INAD and containing tubulovesicular structures, vesicles, mitochondria, and amorphous material; they were distributed both in CNS and PNS, with particular involvement of the gracile and cuneate nuclei of the brainstem (Wada et al., 2009). Iron accumulation was not investigated in these mice. The striking temporal difference in the manifestation of the phenotype among different mouse models is not explained and raises the question whether the presence of the mutant protein may contribute to the development of the disease. Important information regarding the possible pathogenic pathway underpinning the disease have been recently provided by Beck et al. (2011), who performed an in depth morphological and biochemical analysis of the spinal cords and sciatic nerves of *PLA2G6*^-/-^ mice at 15 (presymptomatic stage), 56 (early clinical stage), and 100 weeks (late clinical stage) of age. The presence of PAS-positive granules in the perinuclear space and proximal axons of neurons was the earliest abnormality detected in KO mice. The granules were decorated with antibodies for TOM20, a marker of the inner mitochondrial membrane. The ultrastructure analysis revealed that these granules were remnants of mitochondria filled with dense granules. At later stages swollen axons filled with granules and vacuoles appeared and progressively increased in number with age; many collapsed mitochondrial remnants as well as abnormal mitochondria with branched and tubular cristae and tubulovesicular structures were observed within the swollen axons, where perturbation of the axonal cytoskeleton was also evident. The plasma membranes at axon terminals were also degenerated. The lipid content of these tissues was analyzed by liquid chromatography/electron spray ionization tandem mass spectrometry and by imaging mass spectrometry. A severe perturbation in the relative amount of different types of phospholipids was documented, with increase of the phosphatidylcholine containing AA and docosahexaenoic acid (DHA), decrease of other phosphatidylcholines and increase of phosphatidylethanolamines and cardiolipins. Altogether these data suggest that the absence of iPLA2β enzymatic activity may lead to an insufficient remodeling of the presynaptic and the mitochondrial inner membranes with abnormal accumulation of DHA- and AA-containing phospholipids; these perturbation would cause a progressive degeneration of mitochondria and plasma membranes at synaptic terminals, associated to alteration of the cytoskeleton, and ultimately determining the formation of tubulovesicular structures and swollen axons, the main pathological features of INAD. Given the multi-faceted role of iPLA2β in the cells, the existence of parallel pathways has to be taken into account, as demonstrated by the disturbance in Ca^+^^+^ signaling observed in astrocytes derived from two different mouse models of INAD (Strokin et al., 2012). Exposure of astrocytes from mutant mouse strains to ATP revealed a shorter duration of Ca^+^^+^ responses, associated to reduced capacitative Ca^+^^+^ entry. Similar results were obtained when wild type cells were exposed to a chemical inhibitor of iPLA2β. Defects in Ca^+^^+^ responses could affect the neuron–astrocyte communication and potentially contribute to the development of the disease.

As to the role of iron in PLAN development no studies and data are available. In contrast to PKAN, the presence of iron is not a constant feature of PLAN patients; furthermore, even though the available animal models show a pathology remarkably similar to that observed in patients, they apparently do not show evidence of iron accumulation in the brain. Noteworthy, the analyses were usually limited to the histological detection by the Perls’ staining. Altogether it may well be that the metal has not a relevant role in PLAN pathogenesis, but more careful studies could provide important information as to the possible connection between lipid and membrane homeostasis and iron metabolism in the brain.

### FATTY ACID HYDROXYLASE-ASSOCIATED NEURODEGENERATION

Most of the cases of NBIA are associated to mutations in *PanK2* and in *PLA2G6* genes, but many other NBIA genes have been recently identified (**Table 1**). Interestingly the relevance of lipid metabolism and membrane remodeling is further highlighted by the association of mutations in the gene coding for the fatty acid-2-hydroxylase (FA2H) enzyme (Kruer et al., 2010) with another rare form of NBIA, named FAHN. The same gene is also involved in hereditary spastic paraplegia SPG35 (Dick et al., 2010) and in leukodystrophy (Edvardson et al., 2008). The clinical phenotype is quite similar to that of PLAN with spasticity, dystonia, ataxia and oculomotor disturbances. The MRI shows bilateral hypointensity in the GP and in SN, related to iron accumulation, cortical atrophy, and white matter lesions. The enzyme catalyzes the hydroxylation of fatty acids in sphingolipids, essential constituents of myelin sheaths (**Figure 3**). The relevance of this metabolic pathway for axonal functioning and maintenance is evidenced by the phenotype of *FA2H*^-/-^ mice, showing evident signs of demyelination with axonal enlargement and loss in the CNS (Zöller et al., 2008; Potter et al., 2011).

### MITOCHONDRIAL MEMBRANE PROTEIN-ASSOCIATED NEURODEGENERATION

Mutations in the *C19orf12* gene are now associated with MPAN, an autosomal recessive disorder that represents between 5 and 30% of NBIA cases (Hartig et al., 2011; Panteghini et al., 2012; Hogarth et al., 2013). The evaluation of the genetically confirmed cases of MPAN allowed the identification of a distinctive clinical phenotype characterized by pyramidal and extrapyramidal signs, cognitive decline, neuropsychiatric changes, optic atrophy, and upper and lower motor neuron signs (Hartig et al., 2011; Hogarth et al., 2013). Great variability is present both in disease onset (between 3 and 30 years) and progression. Gait instability or visual impairment are often the initial symptoms, then followed by muscular weakness and atrophy, dystonia, and dysarthria. Almost constant is the cognitive decline leading to dementia as well as the appearance of neuropsychiatric modifications. Brain iron accumulation is the most significant sign at MRI. It usually involves both the GP and the substantia nigra; it can be accompanied by cortical and cerebellar atrophy. The neuropathologic assessment has been performed in two cases, with very similar results and evidence of iron accumulation, axonal spheroids, tau and Lewy pathology that affected the basal ganglia, the archicortex, the neocortex, and the spinal cord. Iron was evident in the GP and the substantia nigra, inside neurons, astrocytes and macrophages with perivascular localization and associated with neuronal loss and gliosis. Numerous eosinophilic axonal spheroids were evident and were strongly immunoreactive for ubiquitin, and less positive for tau or APP staining. Extremely remarkable was the Lewy pathology with the presence of Lewy neurites and Lewy bodies positive for α-synuclein staining in many brain regions. In one case the overall burden was greater than that observed in cases of sporadic Lewy body disease (Hogarth et al., 2013). More recently mutations in *C19orf12* gene have been linked to cases of hereditary spastic paraplegia type 43 (Landouré et al., 2013) and pallido-pyramidal syndrome (Kruer et al., 2014), thus extending the clinical spectrum associated to *C19orf12* gene variants.

The *C19orf 12* gene consists of three exons and codes for two alternative mRNA isoforms (NM_001031726.3 and NM_031448.4) and proteins that differ for the presence of a stretch of 11 amino acids at the N-terminus of the longer form. Different types of mutation have been identified and many of them lead to truncated, non-functional proteins. Among the missense mutation, the c.32C > T is the most common and affects tyrosine 11, exclusively present in the longer form. The protein has a long hydrophobic domain (amino acid residues 42–75) that could represent a transmembrane region; the presence of two prolines in the middle of the domain suggests the possibility of an hairpin structure. The longer form is localized in the mitochondria (Hartig et al., 2011) and possibly in the ER (Landouré et al., 2013); its expression level is more significant in the brain, in blood cells and adipocytes and it appears to be co-regulated with genes involved in fatty acid biogenesis and valine, leucine, isoleucine degradation, that indicates a connection with CoA and lipid metabolism and mitochondria. No other information is available to explain the connection between the protein function, the neurodegenerative process, and iron accumulation in the brain, but very recently a *Drosophila* model with impaired expression of the two orthologs of human C19orf12 was obtained (Iuso et al., 2014); those flies showed reduced life span and climbing activity, signs of neurodegeneration (vacuoles) but not of iron accumulation (negative Prussian blue staining). The model will be an important tool to explore the mechanisms underlining the disorder.

## OTHER FORMS OF NBIA

### β-PROPELLER PROTEIN-ASSOCIATED NEURODEGENERATION

Very recently a subset of NBIA cases with homogenous clinical phenotype and disease history (known as SENDA) has been linked to mutations in the *WDR45* gene (Haack et al., 2012; Saitsu et al., 2013) and is now identified as BPAN. The clinical pattern is characterized by global developmental delay and intellectual deficiency in early childhood then followed by further neurological and cognitive regression in late adolescence or early adulthood, with parkinsonism, dystonia, and sometimes ocular defects and sleep perturbation (Hayflick et al., 2013). At the time of the clinical deterioration the MRI shows clear sign of iron accumulation involving the SN and the GP. A common and distinctive feature on T1-weighted-imaging is the presence of a thin line of hypointensity in the SN and cerebral peduncles, surrounded by a hyperintense halo. Cerebral and sometime cerebellar atrophy are present. The pathology from a single postmortem sample is available (Hayflick et al., 2013) and showed evident iron accumulation in the SN and to a lesser extent in the GP. This was associated with the presence of axonal spheroids, gliosis and neuronal loss. In the cerebellum there was a significant reduction of Purkinje cells. Neurofibrillary tangles were present in different brain regions, while no positivity for α-synuclein or APP deposition was evident. BPAN is due to “*de novo*” mutations in the WDR45 gene causing loss of function of the encoded protein; even though the gene is at the chromosome X, males and females present the same clinical phenotype, which seems to be due to somatic mosaicism or skewing of the X chromosome inactivation (Haack et al., 2012). The *WDR45* gene codes for a protein (WIPI4) with a seven-bladed beta-propeller structure and a phosphoinositide-binding motif for membrane interaction. It belongs to the large family of WD40 repeat protein and is one of the four mammalian homologs of yeast Atg18, an important regulator of autophagy. Its involvement in autophagy has been documented in yeast and mammalian cells (**Figure 3**), where it interacts with ATG2 (Behrends et al., 2010), and in *C. elegans*, where its deletion leads to accumulation of early autophagosome (Lu et al., 2011). The protein amount is clearly reduced in lymphoblast cells from BPAN patients and the autophagosome formation is hindered at an early stage as shown by the accumulation of LC3-II protein and its co-localization with ATG9a in enlarged membrane structures (Saitsu et al., 2013). Even though the relevance of the autophagy process in neurons and brain is well documented, this disorder represents the first direct link between the autophagy machinery and neurodegeneration; it will be of great interest to analyze the correlation with iron homeostasis.

### KUFOR–RAKEB SYNDROME

Also Kufor–Rakeb disease (KRS; OMIM 606693) is due to mutations in a gene, namely *ATP13A2*, encoding a protein involved in degradation processes. It is a rare autosomal disorder also identified as Parkinson’s disease 9 and characterized by early-onset parkinsonism, pyramidal signs, altered eye movements and dementia. Some cases show signs of bilateral iron deposition in the basal ganglia at the MRI, and they can be added to the group of NBIA (Schneider et al., 2010). Mutation in the same gene are also linked to cases of neuronal ceroid lipofuscinosis (NCL; Bras et al., 2012). Indeed, the accumulation of lipofuscin and α-synuclein was also observed in ATP13A2-null mice (Schultheis et al., 2013).

The gene codes for a type 5 P-type ATPase, a cation pump located in the lysosome whose function is not defined yet. Different studies in patients’ primary cells and in *ATP13A2* null-mice have investigated the function of the protein suggesting its involvement in divalent cation handling, and lysosomal and mitochondrial functioning. The analysis of fibroblasts from patients carrying mutations in the *ATP13A2* gene evidenced severe perturbations of lysosomal function, with impaired degradation of substrates, reduced processing of lysosomal enzymes and decreased autophagosomes clearance (Dehay et al., 2012; Usenovic et al., 2012). The downregulation of *ATP13A2* expression in dopaminergic neurons or mouse primary cortical neurons induced the same phenotype and cell death, together with α-synuclein accumulation. Fibroblast from patients’ also showed impaired maintenance of mitochondria, with network fragmentation, mitochondrial DNA alterations, reduced membrane potential, and ATP production (Grünewald et al., 2012). Similarly, silencing of *ATP13A2* in rat primary cortical neurons induced mitochondrial fragmentation either in basal condition or after exposure to cadmium (Ramonet et al., 2012). It is probable that these perturbations in organelle function and structure are linked to altered cation transport and/or homeostasis. Lysosomal Zn^+^^+^ sequestration was shown to be impaired in patients’ fibroblasts (Tsunemi and Krainc, 2013) and olfactory neurosphere cultures (Park et al., 2014) and this was associated with increased expression and accumulation of α-synuclein, elevation of ROS levels, ATP depletion and mitochondrial dysfunction, leading to cell death. The overexpression of ATP13A2 or Zn^+^^+^ chelation could block Zn^+^^+^ toxicity (Park et al., 2014). Furthermore KRS mutants were not able to protect cells form Mn^+^^+^-dependent cytotoxicity (Tan et al., 2011). Even though no evidence suggests a direct role of the P-type ATPase in iron intracellular handling, the lysosomes, and acidic endosomes are fundamental for iron homeostasis and perturbation of their functioning may be linked to the accumulation of the metal observed in the brain of some KRS patients.

## CONCLUSIONS AND FUTURE PERSPECTIVES

While each of these NBIA types is associated to mutations in different genes, it is possible to underline common features that may point to shared pathogenic pathways. Aceruloplasminemia and neuroferritinopathy have in common iron mis-handling, which is the primer trigger of cellular oxidative damage, while the involvement of lipid metabolism and mitochondria is evident in many of the other diseases. It is clear for PKAN and PLAN in which mitochondrial network fragmentation, altered cristae and structural and functional abnormalities are well documented (Rana et al., 2010; Beck et al., 2011; Brunetti et al., 2012) and are probably linked to perturbation in the synthesis (PanK2) or the remodeling (iPLA2β) of membrane lipids. It will be of interest to analyze mitochondria from patients with mutations in *COASY*, that alters the same metabolic pathway of Pank2, and in C19orf12, that is linked to the mitochondrial outer membrane and is presumably connected with lipid metabolism (Hartig et al., 2011). Interestingly enough mitochondrial structure and function perturbations were found also in cells with defects in type 5 P-type ATPase, involved in KRS. This could be due to an impairment of lysosomal activity and ensuing defects in the clearance of damaged mitochondria (mitophagy). Perturbations in this process seem to play a major role in the development of PD, particularly in the genetic forms linked to mutations in Pink1, DJ-1, and Parkin, and this could explain the overlap between some forms of NBIA and PD. Mitochondria are also strategic organelles for the regulation of iron metabolism and significant connection among iron and altered membrane structure (Patil et al., 2013) and mitophagy (Allen et al., 2013) have been described in the literature. In yeast defects in cardiolipin synthesis lead to diminished formation of iron–sulfur clusters (ISC) and iron accumulation in the organelles, a feature often observed in disorders with faulty ISC assembly, such as Friedreich ataxia or X-linked sideroblastic anemia with ataxia (XLSA-A; Rouault, 2012). Interestingly mitochondrial iron overload often brings up cytosolic iron deprivation and a vicious cycle with increased iron uptake and subsequent deposition in mitochondria (Huang et al., 2011). Such an iron misdistribution could be at the same time a strong trigger for the production of ROS, as occurs in aceruloplasminemia and neuroferritynopathy, and hinder mitochondrial renewal (Allen et al., 2013). The interplay among membrane homeostasis, mitochondria functioning, and iron metabolism may thus play a fundamental role in the development of different types of NBIA and further investigation in this field should lead to a better understanding of the biological mechanisms underpinning these neurodegenerative processes.

## Conflict of Interest Statement

The authors declare that the research was conducted in the absence of any commercial or financial relationships that could be construed as a potential conflict of interest.
